# Temporal changes in the bacterial microbiome of the salivary gland and midgut tissues of *Rhipicephalus sanguineus* (s.l.) ticks in South Africa

**DOI:** 10.1038/s41598-025-99189-0

**Published:** 2025-05-20

**Authors:** Rebecca E. Ackermann, Cory A. Gall, Kelly A. Brayton, Nicola E. Collins, Ilana van Wyk, Jeanette Wentzel, Agatha O. Kolo, Marinda C. Oosthuizen

**Affiliations:** 1 Department of Veterinary Tropical Diseases, University of Pretoria, Onderstepoort, South Africa; 2 Department of Veterinary Microbiology and Pathology, Washington State University, Pullman, USA; 3 Hans Hoheisen Research Centre, Faculty of Veterinary Science, University of Pretoria, Hoedspruit, South Africa; 4 Department of Molecular Microbiology and Immunology, The University of Texas at San Antonio, San Antonio, TX USA

**Keywords:** Bacterial microbiome, 16S rRNA, *Anaplasma*, *Coxiella*, Amplicon sequence variants, Dog, Bacterial infection, Entomology

## Abstract

Tick-borne bacterial pathogens from animals play a significant role in the (re)emergence of human diseases. *Rhipicephalus sanguineus* sensu lato, a globally prevalent tick, predominantly parasitises domestic dogs but can also feed on humans. We characterised temporal changes in the bacterial microbiome of the midgut and salivary gland tissues of *R. sanguineus* s.l. ticks and analysed their potential as reservoirs for pathogenic bacteria. A 16 S microbiome and amplicon sequence variant (ASV) approach was used to study the bacteria present in the tissues of *R. sanguineus* s.l. ticks collected from dogs in Hluvukani, a village in a rural community in Bushbuckridge, Mpumalanga, South Africa, in 2016, 2017 and 2019. Post processing, we obtained 43,161 total sequence reads which were clustered into ASVs by sample year. The final ASVs dataset consisted of seven genera: *Coxiella*, *Anaplasma*, *Escherichia/Shigella*, *Ehrlichia*, *Borrelia*, *Rickettsia* and *Wolbachia*. No differences in the microbiome profiles of the MG and SG tissues were noted. *Coxiella* endosymbionts dominated the microbiome in all years. *Anaplasma* was first detected in 2017, and an increase in *Anaplasma* levels was detected in 2019, when compared to 2017. All other genera were present at low levels. With the exclusion of *Wolbachia*, the other detected genera could have pathogenic potential, highlighting the role that *R. sanguineus* s.l. might play as a reservoir of pathogens.

**Supplementary Information:**

The online version contains supplementary material available at 10.1038/s41598-025-99189-0.

## Introduction

Zoonotic pathogens from domestic and wild animals are a leading cause of emerging and re-emerging diseases in humans^1^. Ticks, with their hematophagous feeding behaviours, wide host range, and global distribution are ideal vectors of zoonotic pathogens among animals and humans^2^. Analysing the bacterial microbiome of specific tissues like the midgut (MG) and salivary glands (SG) is an effective method for identifying tick-borne pathogens (TBPs) and endosymbionts^3^. Bacteria from a blood meal will invade the tick’s MG tissues, traverse through the tick cells, and finally infiltrate the SG cells where they can be transmitted during the next blood meal. Thus, the MG and SG are the organs of acquisition and transmission, respectively. Primary endosymbionts significantly impact tick fitness and survival and can influence TBP acquisition and transmission^3,4^.

TBPs pose a substantial risk to rural South African communities at the interface between wildlife, livestock, and humans^1^. *Rhipicephalus sanguineus* sensu lato (s.l.) is a species complex consisting of a tropical and temperate lineage. The tropical lineage is found in countries such as Mozambique, Zambia, Kenya and South Africa, while the temperate lineage is found in countries such as Argentina, Chile, and Italy^5^. However, there is still some uncertainty regarding the phylogenetic relationships within this species complex, thus we will refer to *Rhipicephalus* sanguineus s.l. throughout the study. *Rhipicephalus sanguineus* s.l., the dominant tick species on domestic dogs, is commonly found around human dwellings and can parasitize humans, as well as most vertebrate hosts^6^. This tick transmits *Ehrlichia canis*^7^, and several potentially zoonotic pathogens, including *Babesia canis*, *Rickettsia conorii*^8^, *Rickettsia rickettsii*^9^, and *Rickettsia massiliae*^10^. While *R. sanguineus* s.l. may transmit *Anaplasma platys*, which has been implicated in zoonotic infections, its vector competence for *A. platys* is still under investigation^11^. Furthermore, *R. sanguineus* s.l. is known to harbour a specific *Coxiella* endosymbiont (CE), which can influence tick fitness and bacterial acquisition or transmission^12,13^. Thus, it is possible that *R. sanguineus* s.l. can significantly contribute to the spread of zoonotic diseases in rural communities.

Hluvukani, a rural village situated within the eastern part of Bushbuckridge Local Municipality, Mpumalanga, South Africa, is at a wildlife-livestock-human interface, making it susceptible to tick infestations. Surrounded by conservation areas, community members rely on subsistence farming and most people own livestock^14^, which are kept in close proximity, often in kraals near their homes. Various animals, such as cows, goats, pigs, and chickens, are common, and domestic dogs roam freely, often assisting with hunting and herding. These factors, combined with the proximity to wildlife on neighbouring conservation areas, contribute to frequent contact between the community, their animals, and ticks. This creates an ideal situation for the spread of TBPs between wildlife, livestock and humans. Historically, research in this area has focused on the impact of tick-borne pathogens (TBPs) on livestock, with less attention on human health^15^.

The study analysed temporal changes in the bacterial microbiome of the midgut and salivary gland tissues of *R. sanguineus* s.l. ticks and assessed its potential role in human and animal health in this rural community. A Pacific Biosciences (PacBio) circular consensus sequencing (CCS) approach was used to sequence the 16 S rRNA gene and an amplicon sequence variant (ASV) pipeline was used for the analysis of the microbiome data.

## Materials and methods

### Ethical approvals

The following committees approved this project: The Faculty of Veterinary Science, University of Pretoria (UP) Research Ethics Committee (REC010-18), the UP Animal Ethics Committee (V012-18 and V064-16), and the UP Faculty of Humanities Research Ethics Committee (GW20180719HS). Permission was obtained to conduct the research, in terms of Section 20 of the Animal Diseases Act of 1984, from the Department of Agriculture, Land Reform and Rural Development (DALRRD) South Africa, with reference numbers 12/11/1/1/8 and 12/11/1/1. All experiments in this manuscript were performed in accordance with the relevant guidelines and regulations. This study is reported in accordance with ARRIVE guidelines.

### Sample collection and genomic DNA extraction

The study aimed to understand how the bacterial microbiome of *R. sanguineus* s.l. impacted human health in a rural community. Ticks were collected from dogs from areas surrounding Hluvukani (24.644397° S, 31.347644° E), a village in the eastern part of the Bushbuckridge local Municipality, Mpumalanga, South Africa, in 2016, 2017, 2018 and 2019. Dogs were sampled at random, with inclusion based on factors like the weight, health, and dog hostility as well as owner presence. Dogs weighing less than 500 g (or younger than 6 weeks) or showing clinical symptoms of disease or injury were not included, to avoid any additional stress during sampling, as indicated by the ethics committees. If multiple dogs were present at a household, all were examined for ticks. As a pilot study, a total of ten dogs were sampled in 2016 and a total of ten dogs were sampled in 2017. From 2018 to 2019, a total of 73 dogs were sampled. As sampling was random, and dogs within this community are usually able to roam freely, it is plausible that the same dog was sampled more than once. Ticks were collected using blunt forceps, focusing on male *R. sanguineus* s.l. ticks due to the degeneration of certain organs in engorged females^16^. Collected ticks were stored in a temperature and humidity-controlled chamber for 2 days to allow for the blood meal to be cleared from the midgut to prevent false-positive detection of tick infection^3,17–19^ at the Hans Hoheisen Research Centre. Ticks were morphologically identified using a tick identification key^20^ and surface sterilized using a triple tick rinse^21^.

Ten male *R. sanguineus* s.l. ticks per dog were dissected using surface sterilized tweezers and Hyde single-edged razor blades (Hyde, Massachusetts, United States)^21^. The ten SGs comprised one pool, while the ten MGs comprised a second pool. Each pool was placed into a storage solution with Cell Lysis Solution (Qiagen, Valencia, CA) and proteinase K (1.25 mg/mL) (Thermo Fisher Scientific, Massachusetts, USA), and genomic DNA was extracted using the PureGene Extraction kit (Qiagen) according to the manufacturer’s specifications. It was not always possible to identify ten *R. sanguineus* s.l. ticks from each dog, and SG and MG pools from fewer ticks did not yield sufficient good quality DNA.

### PCR and sequencing

The 16S rRNA gene (V1-V8) was amplified in triplicate, using modified universal barcoded 16S rRNA primers; 27F (5’-AGA GTT TGA TCM TGG CTC AGA ACG-3’) and 1435R (5’-CGA TTA CTA GCG ATT CCR RCT TCA-3’)^3^, targeting an ~ 1300 bp region. In 2016–2017 amplification was performed with Platinum Pfx, following the protocol outlined by Gall^3^. In 2019, due to the discontinuation of Platinum Pfx, Phusion Flash High-Fidelity PCR Master Mix (Thermo Fisher Scientific) was used, following the manufacturer’s protocol. Cycling conditions were: initial denaturation at 98 °C for 30 s, 40 cycles of 98 °C for 10 s, 60 °C for 30 s, and 72 °C for 30 s, with a final extension at 72 °C for 10 min. Triplicate PCR products were pooled, and DNA concentration was measured with a bioanalyzer (Agilent, Santa Clara, CA, USA). Pooled PCR products were submitted to the Washington State University’s Sequencing Core where the samples were pooled in equimolar amounts and CCS was conducted on the PacBio Sequel system (Pacific BioSciences, Menlo Park, CA). The raw microbiome datasets were deposited in the National Centre for Biotechnology Information (NCBI) Sequence Read Archive (SRA) under bioproject accession number PRJNA1176486.

### Amplicon sequence variant analysis

The Dada2 PacBio pipeline, created specifically for long sequence reads was used for quality filtering and processing of the raw sequence data^22^. The analysis was implemented in R^23^ and R studio^24^, using the following R packages: Dada2 package^25^ (version 1.30.0), Biostrings package^26^ (version 2.70.1), ShortRead package^27^ (version 1.60.0), and Reshape2 package^28^ (version 1.44.4).

Raw sequencing reads from different runs were analysed individually on the Dada2 pipeline. Quality filtering removed low-quality sequences or reads with high expected errors, retaining sequences between 1250 bp and 1600 bp. Sequences were dereplicated, run through an error model, denoised and chimeras removed, to produce ASVs. Taxonomic and species assignments were made using the RefSeq + RDP taxonomic training dataset formatted for Dada2^29^. The RefSeq + RDP dataset incorporates the RefSeq database from NCBI and supplements with the RDP dataset, to provide high taxonomic resolution. The output was further analysed using the R phyloseq package (version 1.46.0).

To identify possible contaminants, a database was generated using information from previous studies^30–36^ (Supplementary file 1: Table 1). The obtained ASVs were filtered to remove contaminant taxa and those not identified at the genus level. A rarefaction curve was performed using the iNEXT package^37^ (version 3.0.1), which was designed to perform rarefaction curves without needing to down-sample (rarefy) the data. The final ASV data was visualised on the R ggplot2 package^38^ (version 3.4.4) and R gridExtra package^39^ (version 2.3), to create a 100% stacked bar plot containing the abundance of each ASV per sample.

For ASVs not identified to the species level, a nucleotide NCBI BLAST search using blastn^40^ was performed to identify the closest match in GenBank. The association between the number of reads of *Anaplasma* and *Coxiella* in all samples was assessed using both the Kendall Correlation and Spearman Correlation tests using the R stats package (version 4.3.2). A comparison between the microbiome profiles of corresponding MG and SG tissues from the same host (where available) was performed and visualised with a heatmap using the R package pheatmap (version 1.0.12).

### Phylogenetic analysis

ASVs from organisms of interest (i.e. *Coxiella*, *Anaplasma*, *Ehrlichia*, *Rickettsia-*like and *Borrelia*-like) were aligned with homologous sequences from GenBank using QIAGEN CLC Main Workbench 24.0 (QIAGEN, Aarhus, Denmark). Information for all 16 S rRNA gene sequences retrieved from GenBank is shown in Supplementary Tables 2–6. The 16 S sequences reported in this study were deposited in GenBank under the following accession numbers: *Wolbachia-like* endosymbiont: PQ508350; *Ehrlichia canis*: PQ508351; *Borrelia-like*: PQ508352; *Rickettsiales*: PQ508353; *Coxiella* endosymbiont: PQ508354, PQ508355; *Anaplasma centrale*: PQ508356, PQ508357, PQ508358, PQ508359, PQ508360, PQ508366, PQ508362, PQ508363, PQ508364, PQ508365; *Anaplasma platys*: PQ508361. Alignments were truncated as follows: *Coxiella* (1124 bp, 25 sequences), *Anaplasma* (1243 bp, 37 sequences), *Ehrlichia* (1251 bp, 21 sequences), *Rickettsia* (1251 bp, 24 sequences), and *Borrelia* (1297 bp, 23 sequences). Statistical selection of the best-fit model was performed using IQTREE ModelFinder^41^. Maximum likelihood (ML) analysis was performed on the alignments using the IQTREE web server^42^ with ultrafast bootstrap replicates (UF). The models used were: *Coxiella* – K2P + I + G4 (1000 UF), *Anaplasma* - TPM2u + F + I + G4 (3000 UF), *Ehrlichia* - TIM3 + F + I + G4 (1000 UF), *Rickettsia* - GTR + F + I + G4 (1000 UF) and *Borrelia* - TIM3 + F + I + G4 (1000 UF). The ML output was visualised using ITOL: Interactive Tree of Life (version 6.8.1)^43,44^ with ML bootstrap values above 75 shown on the tree, and values above 95 considered good support.

## Results

### Sample collection

The following tick genera were observed co-feeding on the dogs: *Rhipicephalus* (*R. sanguineus* s.l., *R. simus*, *R. turanicus*, *R. microplus*), *Amblyomma* (*A. hebraeum*), *Haemaphysalis* (*H. leachi*) and *Hyalomma* (*Hy. truncatum*). Tick species prevalence was not analysed, however, *R. sanguineus* s.l. was the most predominant tick observed on the dogs in all three years. All dog samples from 2018 and 17 dog samples from 2019 were excluded from further analysis, as they contained too few ticks to yield high quality DNA. *R. sanguineus* s.l. ticks from ten dogs in 2016 and ten dogs in 2017 were used for further analysis, while in 2019, *R. sanguineus* s.l. ticks from six dogs were used for further analysis.

### Sequence analysis

A total of 20 tick pools (10 MG and 10 SG) were processed in 2016, 20 tick pools (10 MG and 10 SG) were processed in 2017, while 12 tick pools (six MG and six SG) were processed in 2019. A subset of samples failed amplification in 2016 (three MG samples and two SG samples), 2017 (six MG samples and five SG samples) and 2019 (one MG sample and one SG sample). These samples were subsequently removed from further analysis. A rarefaction curve analysis indicated that the sequencing effort was sufficient for most samples to capture the microbial diversity present in the samples as evidenced by the plateauing of the curves (Supplementary file 1: Fig. 1). This suggests that additional sequencing would likely yield few new species.

**Fig. 1:**
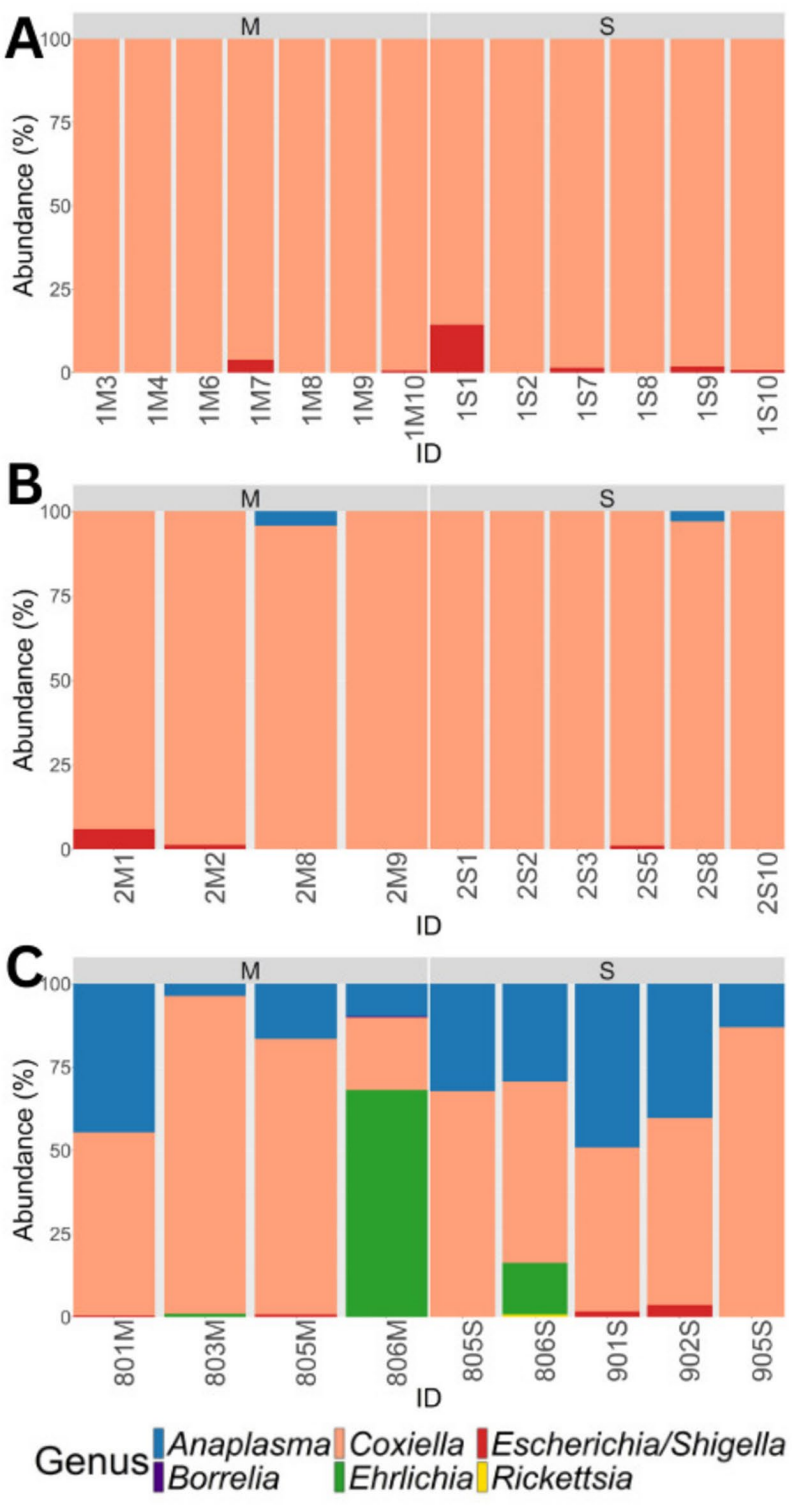
Prevalence and diversity of the bacterial genera of midgut and salivary gland pools of *Rhipicephalus sanguineus* s.l. ticks sampled from domestic dogs in the study site. (**A**) 2016, (**B**) 2017 and (**C**) 2019. Sample identification codes are indicated on the x-axis (M-Midgut pool, S-salivary gland pool, Y1-2016, Y2-2017, Y3-2019).

Samples from 2016 contained 38,241 raw sequence reads (Supplementary file 1: Table 7). After quality control, 58 ASVs were detected from 17,027 sequence reads, assigned to nine genera. After filtering and removing contaminants and two SG samples with low read counts, the microbiome profile included one *Coxiella* ASV (98.23%) found in all samples, and seven *Escherichia/Shigella* ASVs (1.77%), in six samples (Fig. 1A). Samples from 2017 contained 15,642 raw sequence reads (Supplementary file 1: Table 7). After quality control, 39 ASVs were detected from 8,556 sequence reads, assigned to 15 genera. After filtering and removing contaminants, the microbiome profile consisted of one *Coxiella* ASV (98.17%) in all samples, five *Escherichia/Shigella* ASVs (1.16%) in three samples, and one *Anaplasma* ASV (0.67%) in two samples (Fig. 1B). In 2019 samples contained 80,695 raw sequence reads (Supplementary file 1: Table 7). After quality control, 379 ASVs detected from 54,583 sequence reads, were assigned to 106 genera. After filtering, removing contaminants and two MG and one SG low read samples, the microbiome profile consisted of two *Coxiella* ASVs (54.21%) from all samples, five *Escherichia/Shigella* ASVs (0.63%) in four samples, 11 *Anaplasma* ASVs (27.99%) in all samples, two *Ehrlichia* ASVs (17.06%) in three samples, one *Borrelia*-like ASV (0.07%) in one sample and one *Rickettsia*-like ASV (0.04%) in one sample (Fig. 1C).

Species-level assignment indicated that *Coxiella* ASV 1, detected in all three years, was identical to the sequence of the 16 S rRNA gene of a known *Coxiella*-like endosymbiont (CLE) (MZ836861) identified from an *R. sanguineus* s.l. tick. *Coxiella* ASV 2, detected in 2019, was not identified to the species level but had 99.92% identity to a CE from an *R. microplus* tick (CP094229.1). *Escherichia/Shigella* ASVs indicated that all ASVs were similar to 16 S rRNA gene sequences from various known *Escherichia coli* strains (MT647245.1, CP053607.1, CP142044.1)^45,46^. *Anaplasma* ASVs were divided into two groups. Group one (*Anaplasma* ASV 11), detected in two samples from 2017 and one sample from 2019, was identical to a known *A. platys* sequence (CP046391.1), isolated from a dog^47^. Group two (*Anaplasma* ASV 1 to *Anaplasma* ASV 10) detected in 2019 matched *A. centrale. Anaplasma* ASV 1 was identical to the 16 S rRNA gene sequence of the Israel strain of *A. centrale* (CP001759.1)^48^. *Anaplasma* ASV 2 to *Anaplasma* ASV 10 were not assigned to species level. A BLAST search indicated that these ASVs matched *A. centrale* (CP001759.1) with an average of 99.68% identity.

*Ehrlichia* ASV 1 could not be assigned to species level using Dada2, however, a BLAST search identified it as identical to a known *Wolbachia* endosymbiont (MN383047.1) from mosquitoes. *Ehrlichia* ASV 1 will hereafter be referred to as *Wolbachia* ASV1. *Ehrlichia* ASV 2 was identical to *E. canis* (MK507008.1) isolated from a dog and an uncultured *Ehrlichia* (JN121380). *Borrelia*-like ASV 1 was not assigned to species level; the highest BLAST match (98.81% identity) was to an uncultured bacterium (EU137037.1)^49^, with 58% query coverage, and it also matched “*Candidatus* Borreliella tachyglossi*”* (CP025785.1) with 85.69% identity and 100% query coverage. *Rickettsia*-like ASV 1 was not assigned to species level, however a BLAST search indicated that the highest match (91.24% identity), was to an uncultured *Rickettsiales* bacteria (MK616428.1) from a nucleariid amoeba (*Pompholyxophrys punicea*) sampled from a lake in Zwönitz, Germany^50^.

Kendall Correlation and Spearman Correlation tests found a moderate positive association between the number of *Anaplasma* sequence reads and *Coxiella* sequence reads. This was, however, not statistically significant (Kendall: *R* = 0.24, *p* = 0.36. Spearman: *R* = 0.3, *p* = 0.37. (Supplementary file 1: Fig. 2). A comparison between the microbiome profiles of the tissue types (MG and SG) from the same host was visualised on a heatmap (Supplementary file 1: Fig. 3) and no difference could be seen between the tissue types. Due to the small sample size, robust statistical testing could not be performed.

#### Phylogenetic analysis

**Fig. 2:**
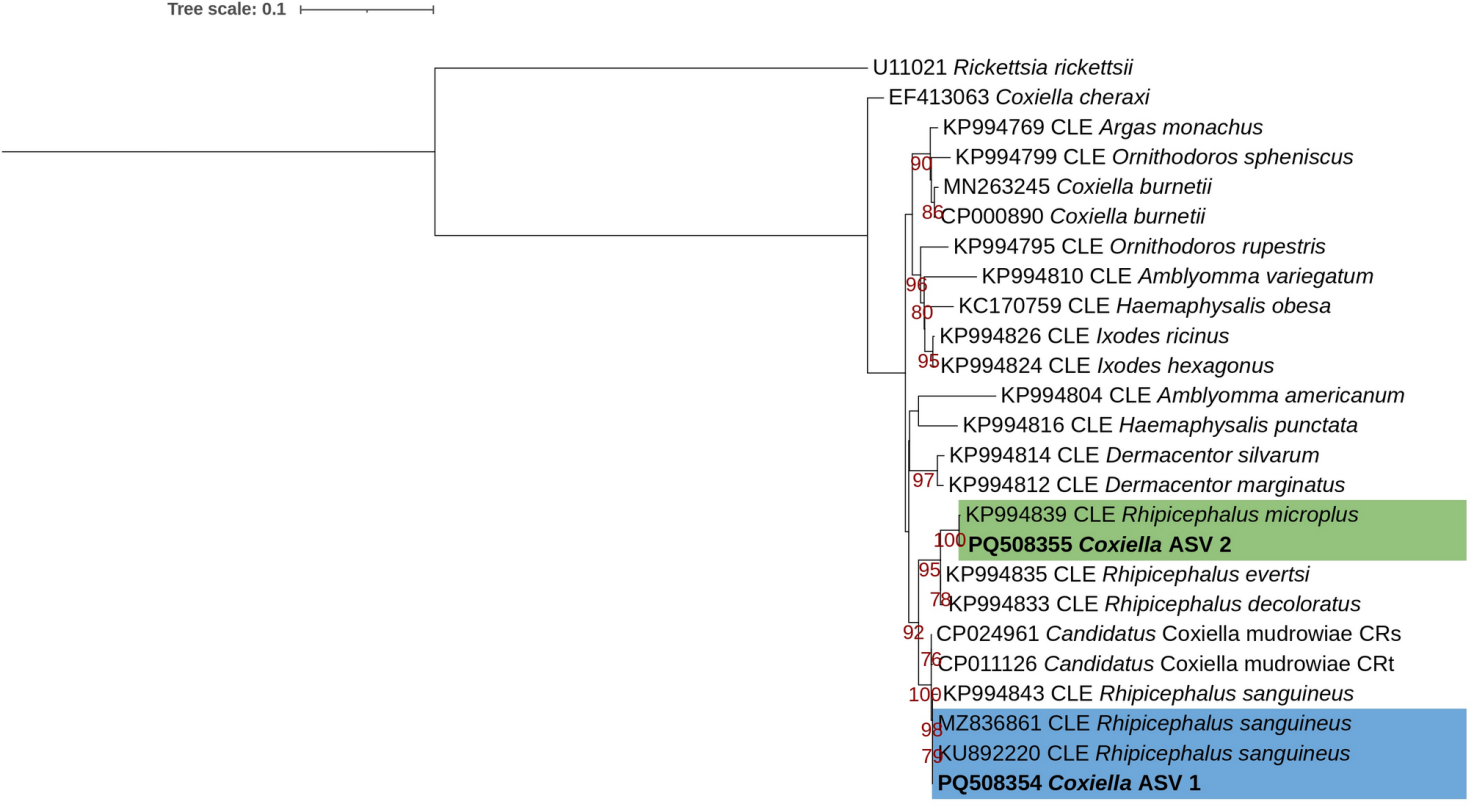
Maximum likelihood analysis indicating the relationships of *Coxiella* amplicon sequence variants detected in this study with known *Coxiella* sequences from GenBank. A maximum likelihood inference was conducted using the K2P + I + G4 model and 1000 ultrafast bootstrap replicates (alignment length 1124 bp). Maximum likelihood bootstrap values (> 75) are indicated below the branch. *Coxiella* amplicon sequence variants are indicated in bold. The *Coxiella* sequences associated with *Rhipicephalus sanguineus* s.l. and *Rhipicephalus turanicus* are indicated in a blue box, while the *Coxiella* sequences associated with *Rhipicephalus microplus* are indicated in a green box. *Rickettsia rickettsii* (U11021) was used as an outgroup.

A ML analysis of the *Coxiella* ASVs (Fig. 2) indicated that *Coxiella* ASVs clustered in a monophyletic group with known CE sequences from *Rhipicephalus* ticks, but with low bootstrap support. This monophyletic group split into two subgroups with moderate bootstrap support (92). One subgroup contained *Coxiella* ASV 1 and sequences from known CEs isolated from *R. sanguineus* s.l. (CP024961, KU892220, MZ836861 and KP994843)^51–53^ and “*Candidatus* Coxiella mudrowiae” from *R. turanicus* (CP011126)^54^. The second subgroup contained *Coxiella* ASV 2 and sequences from known CEs isolated from *R. decoloratus* (KP994833), *R. microplus (*KP994839) and *R. evertsi* (KP994835)^51^. Within this group, *Coxiella* ASV 2 and the CE from *R. microplus* clustered together (99.92% identity), with good bootstrap support (100).

**Fig. 3:**
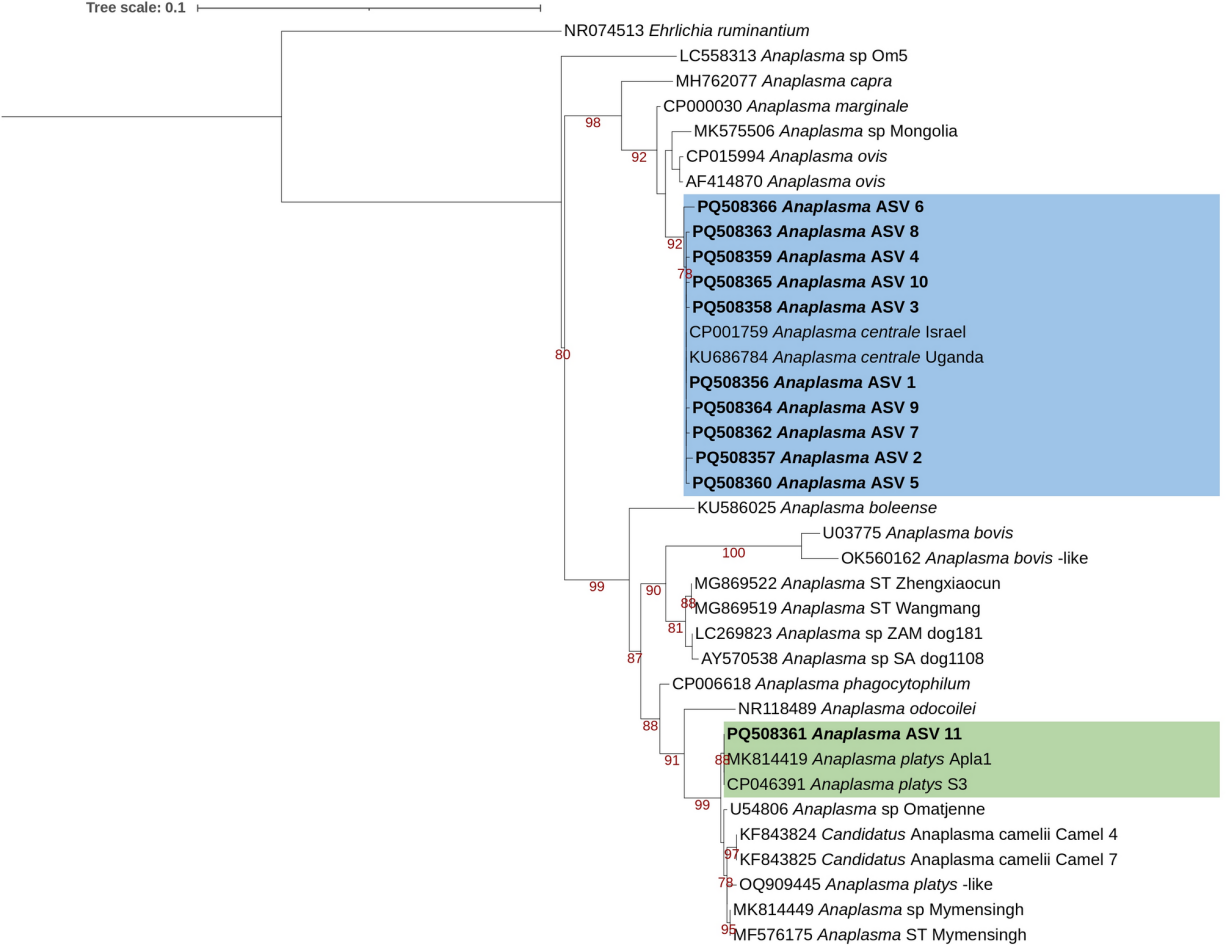
Maximum likelihood tree indicating the relationships of the *Anaplasma* amplicon sequence variants detected in this study, with known *Anaplasma* sequences from GenBank. A maximum likelihood inference was conducted using the TPM2u + F + I + G4 model and 3000 ultrafast bootstrap replicates (alignment length 1243 bp). Maximum likelihood bootstrap values (> 75) are indicated below the branch. *Anaplasma* amplicon sequence variants are indicated in bold. The *Anaplasma centrale* group is indicated in a blue box, while the *Anaplasma platys* group is indicated in a green box. *Ehrlichia ruminantium* strain Welgevonden (NR074513) was used as an outgroup.

A ML tree (Fig. 3) of the 11 *Anaplasma* ASVs, indicated that *Anaplasma* ASV 1 to ASV 10 formed a monophyletic group with two previously characterised *A. centrale* sequences (*A. centrale* Uganda: KU686784 and *A. centrale* Israel: CP001759)^48^, with bootstrap support of 92. *Anaplasma* ASV 11 formed a monophyletic group with two known *A. platys* sequences: *A. platys* S3 (CP046391)^47^ and *A. platys* Apla1 from a dog in the study community (MK814419)^55^, with high bootstrap support of 99.

*Wolbachia* ASV 1 grouped with known *Wolbachia* endosymbionts (MN383047 and KX155506)^56^ and an uncultured bacterium (JX669531) with bootstrap support of 100. It was most closely related to a *Wolbachia* endosymbiont from *Aedes aegypti* (MN383047) (Supplementary file 1: Fig. 4). *Ehrlichia* ASV 2 grouped with known *E. canis* and uncultured *Ehrlichia* sequences (MK507008, NR118741 and JN121380)^57,58^ with bootstrap support of 100. ML phylogenetic analysis indicated that *Rickettsia*-like ASV 1 was ancestral to known *Rickettsia*, *Orientia* and *Occidentia* sequences and clustered together with uncultured bacterial sequences, but with low bootstrap support (Supplementary file 1: Fig. 5). *Borrelia*-like ASV 1 did not group with known *Borrelia* sequences or closely related spirochetes (Supplementary file 1: Fig. 6). Instead, it branched off from the *Cristispira* genus, with good bootstrap support of 96.

## Discussion

This study showed that the bacterial microbiome of MG and SG tissues from *R. sanguineus* s.l. ticks changed over the study period, and highlighted the role that *R. sanguineus* s.l. ticks might play as reservoirs of potential pathogens.

*Rhipicephalus sanguineus* s.l. was the most abundant tick species observed on the dogs in all three years of our study, in agreement with previous findings^59^. As *R. sanguineus* s.l. ticks are well adapted to human dwellings and rely on dogs as their main host, infestations can become extremely high with a consequent increase in the risk of exposure to TBPs for animals and humans^60^. Dogs weighing less than 500 g or younger than 6 weeks (puppies) and showing signs of injury or clinical disease were not sampled, in accordance with ethical approvals. Exclusion of dogs with clinical symptoms could prevent the detection of potential canine bacterial pathogens, however exclusion of puppies should not significantly influence our findings.

In the MG and SG microbiomes of *R. sanguineus* s.l. ticks collected in the study, we found *Coxiella*,* Anaplasma*,* Ehrlichia¸ Escherichia*, *Rickettsia*-like and *Borrelia*-like 16 S rRNA gene sequences. The microbiomes were initially dominated by a CE in 2016 and 2017, consistent with other studies of *R. sanguineus* s.l. tick microbiomes from northern Spain, France, Senegal, Arizona and Oklahoma^61–63^. In 2017, *A. platys* was introduced into the microbiome. By 2019, *Anaplasma* species had increased, with the introduction of large numbers of *A. centrale* sequences. Other pathogens (*Ehrlichia*,* Rickettsia*-like and *Borrelia*-like) emerged in 2019, but at very low levels. Our findings align with Portillo et al.^62^, who also found low levels of *Rickettsia*,* Borrelia*, *Ehrlichia* and *Wolbachia* in *R. sanguineus* s.l. ticks. The difference in the microbiome between 2017 and 2019 could possibly be attributed to climatic differences with a long drought ending in 2019.

The low humidity conditions during a period of drought are unfavourable for tick populations^64^. Therefore, we surmise that the drought led to a decrease in the overall tick population and thus to decreased transmission of bacterial organisms within the community. Once the drought ended, conditions were favourable for tick population expansions allowing for increased transmission of bacterial organisms and changes in the bacterial microbiome.

Another potential explanation for the difference in the microbiome profile could be the change in reagents between 2017 and 2019. PCR reagents have varying levels of amplification efficiency and accuracy which might result in differential amplification of the bacteria present in the samples. The dog host could also potentially influence the changes seen in the microbiome profiles between the sampling years. Dogs were sampled randomly during each sampling excursion, and given that dogs can roam freely within the community, identification of individual dogs was not possible. This introduces variability in the host-related factors, such as diet and environmental exposures, which could all impact the microbiome of *Rhipicephalus sanguineus* s.l.

This study identified a sparse microbiome profile, with an average of 3.3 species in the MG samples and 3 species in the SG samples. These findings align with previous research^65^, which demonstrated that MG and SG samples exhibit lower alpha diversity compared to whole tick samples. This is likely because MG and SG contain tissue-specific microbiomes, whereas whole tick samples can include the MG, SG, Malpighian tubes, haemolymph, heart, tracheae, rectal sac, synganglion and other tissues, leading to a greater variety or total number of bacterial species. Additionally, MG and SG samples are characterized by low biomass, containing a smaller proportion of total bacterial DNA, making them more susceptible to the over amplification of environmental contaminant DNA. In 2019, we detected many potentially contaminating sequences. Given the sensitivity of next-generation sequencing, microbiome studies are prone to external and cross-contamination^66^, warranting the need for negative controls, however this was not part of our standard protocol when this study began. A list of bacterial genera identified as possible contaminants in microbiome studies were removed from the dataset (Supplementary file 1: Table 1). This list included *Bacillus*, which was previously detected in the *R. sanguineus* s.l. tick microbiome^61^, but because it has also been found in laboratory reagents, it was removed as a possible contaminant. This allowed for the 2019 dataset to be compared in a more meaningful way to the 2016 and 2017 dataset.

In this study, CE sequences were detected in every MG and SG sample over three years. Historically, the genus *Coxiella* included only *Coxiella burnetii*, the agent of Q-fever, which is endemic in South Africa, with a prevalence of up to 59% in vulnerable communities^67^. More recently, various CEs have been discovered, mainly in ticks, but also in the spleens of some wild mammals^68^. CEs have evolved with their tick hosts, causing different CEs to appear to be specific for different tick species^53^. These CEs benefit ticks by enhancing immunity and nutrition^12^, and can affect pathogen acquisition and transmission^13^. Molecular methods for detection of *C. burnetii* may cross-react with CEs, potentially overestimating *C. burnetii* infection rates^69^. As *R. sanguineus* s.l. can bite humans, CE transmission to humans might contribute to Q-fever positive results in South Africa, while not manifesting disease. Two *Coxiella* ASVs were present in the 2019 datasets: ASV 1, identical to the known CE from *R. sanguineus* s.l. ticks, and ASV 2, closely related (99.92% identity) to a CE from *R. microplus* ticks. The presence of *Coxiella* ASV 2 could be due to co-feeding of different tick species at the same site on the dog host, or potentially an *R. microplus* tick could have been erroneously included in one of our 2019 pools. To rule this out, we performed molecular typing on the 2019 ticks, which confirmed they were *R. sanguineus* s.l. (data not shown).

*Anaplasma* sequences were detected only in 2017 and 2019. Analysis revealed two species: *A. platys* and *A. centrale*. In 2017, *A. platys* was found in two samples, while in 2019, *A. platys* was found in a single sample, and *A. centrale* was detected in all nine samples. *Anaplasma platys* causes canine thrombocytopenia in dogs^70^, and has been reported in humans^71,72^. Arraga-Alvarado et al.^72^ provided the first clinical evidence of *A. platys* infection in a human patient from Venezuela who exhibited fever symptoms as well as headache and thrombocytopenia. Though studies on the vector competence of *R. sanguineus* s.l. for *A. platys* are limited, *R. sanguineus* s.l. is thought to transmit *A. platys*, since their geographical distributions overlap, and dogs are the primary hosts for both *R. sanguineus* s. l and *A. platys*^73^. Although Simpson, et al.^11^ found no detectable *A. platys* in *R. sanguineus* s.l. fed on laboratory-infected dogs, Snellgrove et al.^74^ showed that *R. sanguineus* s.l. could maintain *A. platys* through transovarial, transstadial, and horizontal transmission, under laboratory conditions. Detection of *A. platys* in one MG and two SG samples of *R. sanguineus* s.l. in our study highlights the possible role of *R. sanguineus* s.l. as a vector.

An unexpected finding was the detection of *A. centrale* in 2019, as dogs are not known hosts, nor is *R. sanguineus* s.l. a known vector. *Anaplasma centrale* has been shown to be transmitted by *Rhipicephalus simus* and *Dermacentor andersoni*^75–77^ and causes a less virulent form of bovine anaplasmosis^78^. A previous study identified *A. centrale* in the SG of *R. sanguineus* s.l. ticks but did not demonstrate transmission to calves^77^.

The introduction of *A. platys* and *A. centrale* into the *R*. *sanguineus* s.l. microbiome in 2017 and 2019, respectively, could be from wildlife or cattle. While there is little information regarding the role of wildlife in the epidemiology of *Anaplasma* in Africa, *A. platys* and *A. centrale* have been documented in African buffalo^79–82^, and *A. centrale* has also been documented in black and blue wildebeest, eland, waterbuck, zebra, warthog, and lion^78,82^. The introduction of these organisms into adult *R*. *sanguineus* s.l. ticks could occur through transstadial transmission from immature ticks feeding on smaller wildlife hosts. This emphasizes the significance of *R. sanguineus* s.l. at the wildlife-livestock-human interface. Another source could be the movement of cattle and dogs within the community. During sampling, we observed livestock being moved between residences, grazing areas, and dip tanks, with dogs often accompanying them as herding dogs. This activity could facilitate the exchange of ticks and thus TBPs among domestic animals throughout the community.

The introduction of *Anaplasma* coincided with a moderate increase in *Coxiella*, however a Spearman and Kendall Rank correlation indicated that this correlation was not statistically significant. These preliminary findings warrant further study into the correlation between these bacteria in *R. sanguineus* s.l. It is also important to mention that while the proportion of a given bacterium must change with the introduction of additional species, 16 S microbiome sequencing does not indicate whether the absolute numbers of the bacterium have changed significantly.

This study also detected *Escherichia/Shigella* sequences in various samples across all three years. *Escherichia/Shigella* has been detected in several bacterial tick microbiome studies^83–85^. All variants of *Escherichia/Shigella* were similar to known *E. coli* strains, however there is currently no evidence to suggest that *E. coli* is transmitted by *R. sanguineus* s.l.

In two 2019 samples, we detected *Ehrlichia* ASVs. One sequence present at low read numbers (*Ehrlichia* ASV 2) was classified as *E. canis*, the etiological agent of canine monocytic ehrlichiosis. *Rhipicephalus sanguineus* s.l. is a known vector of *E. canis*, and *E. canis* has been previously detected in *R. sanguineus* s.l. ticks as well as in dogs in the community^86,87^. While *E. canis* has not been documented in humans, it does infect wild canids^88^.

During the ASV-based taxonomy assignment, a second “*Ehrlichia* variant” (*Ehrlichia* ASV 1) was detected. However, BLAST search and phylogenetic analysis revealed it to be identical to a *Wolbachia* endosymbiont from a mosquito. The ASV-based taxonomic assignment utilizes an offline database that is not as robust and complete as an online method such as BLAST. No *Wolbachia* sequences were present in the offline database and thus identification was made as *Ehrlichia*, a very closely related genus.

While ticks harbour endosymbionts like *Coxiella*, little is known regarding *Wolbachia* in ticks. One study found *Wolbachia* in *Ixodes* ticks due to parasitism by *Ixodiphagus hookeri* (a tick wasp) harbouring a *Wolbachia* endosymbiont^89^. In our study we saw high *Wolbachia* read numbers in one MG sample with lower numbers in the corresponding SG sample, and a second positive MG sample, which suggests that this was not an accidental finding. Further investigation is needed to determine the origin of this *Wolbachia* variant.

In 2019, we detected very low read counts of a *Borrelia*-like sequence from a single MG sample. Neither the ASV method nor a manual NCBI BLAST search could identify the sequence to species level, and phylogenetic analysis also failed to determine its relationship to known *Borrelia* sequences. This sequence might belong to an unclassified genus within the *Borreliaceae* family (closest match is 85.69% identity to “*Candidatus* Borreliella tachyglossi”), or it could be a chimeric sequence, as each half of the sequence has a higher match (> 94%) to different uncultured bacteria. We detected the *Borrelia*-like sequence in a single midgut sample, which is in alignment with previous research which found that *Borrelia* species are “stored” in the midgut until a second blood meal is taken, upon which they move into the salivary glands^90^.

A *Rickettsia-*like sequence was detected in a single SG sample from 2019 at very low read counts. Further investigation could not identify the sequence to species level, using either the ASV method or a manual BLAST search. The highest match was to an uncultured Rickettsiales bacterium, an endosymbiont of the amoeba, *Pompholyxophrys punicea*^50^. Phylogenetic analysis indicated that it clustered with 16 S rRNA gene sequences from various uncultured Rickettsiales, some from amoebas and others from environmental samples. Further investigation is needed to determine the occurrence and importance of this *Rickettsia*-like organism.

This study had several limitations that should be taken into consideration. Firstly, no negative “blank” controls were included during sample preparation and sequencing, making it difficult to identify potential contaminants. Secondly, during the study certain PCR reagents became unavailable, which led to protocol alterations between years, potentially affecting the microbiome profile. Thirdly, the study had a small sample size due to the difficulty in collecting enough adult male *R. sanguineus* s.l. ticks from the community dogs. Lastly, samples were analysed from non-consecutive years (samples from 2018 did not yield high quality DNA), creating a data gap between 2017 and 2019, which may have resulted in valuable information on the introduction of *Anaplasma* not being analysed. Advantages of our study include the use of dissected organs (allows for a cleaner look at the actual tick microbiome rather than environmental species that cling to the surface of the tick), use of pools (which provide a better grasp of the overall population, and reduces the variation seen when analysing individual ticks) and our longitudinal study demonstrates how the tick microbiome varies over time.

## Conclusion

Our study revealed the bacterial microbiome diversity of *R. sanguineus* s.l. MG and SG tissues from dogs from a rural community in Mpumalanga, South Africa. We identified *R. sanguineus* s.l. ticks as potential reservoirs for pathogens like *A. platys* and *A. centrale*, though further research is needed to confirm their reservoir role and vector competence. Our findings also suggest that *R. sanguineus* s.l. from the community hosts a species-specific CE, corroborating earlier findings in *R. sanguineus* s.l. ticks^51^. We observed a potential correlation between *Coxiella* and *Anaplasma*, warranting further investigation. Additionally, while other pathogenic bacteria were detected, their impact remain unclear. Overall, our research highlights the role of *R. sanguineus* s.l. in influencing pathogen diversity and distribution at the wildlife-livestock-human interface in rural South Africa.

## Electronic supplementary material

Below is the link to the electronic supplementary material.

Supplementary Material 1

## Data Availability

The raw microbiome datasets generated during the current study are available in the NCBI SRA (PRJNA1176486, https://www.ncbi.nlm.nih.gov/sra/?term=PRJNA1176486). 16 S sequences reported in this study are available on GenBank under accession numbers: Wolbachia-like endosymbiont: PQ508350. Ehrlichia canis: PQ508351. Borrelia-like: PQ508352. Rickettsiales: PQ508353. Coxiella endosymbiont: PQ508354, PQ508355. Anaplasma centrale: PQ508356, PQ508357, PQ508358, PQ508359, PQ508360, PQ508362, PQ508363, PQ508364, PQ508365, PQ508366. Anaplasma platys: PQ508361.
