# Supplementary material for: Temporal changes in the bacterial microbiome of the salivary gland and midgut tissues of Rhipicephalus sanguineus (s.l.) ticks in South Africa
Source: Sci Rep. 2025 May 20;15:17434. doi: 10.1038/s41598-025-99189-0 (PMC12092631; doi:10.1038/s41598-025-99189-0)
Supplement: Supplementary file 1 — Supplementary Material 1 [file 41598_2025_99189_MOESM1_ESM.docx]

# Supplementary file 1:

**Table 1:** List of contaminating genera identified in this study based on information from previous studies on possible bacterial contaminants.

| Genus | Habitat | Genus | Habitat | Genus | Habitat |
| --- | --- | --- | --- | --- | --- |
| *Acidibacter* | Water | *Ezakiella* | Human | *Nocardioides* | Soil |
| *Aciditerrimonas* | Soil | *Flavobacterium* | Water | *Nosocomiicoccus* | Hospital surfaces |
| *Acidovorax* | Water/environment | *Fusobacterium* | Human | *Ornithinimicrobium* | Green algae |
| *Acinetobacter* | Animal/environment | *Gemella* | Human | *Panacagrimonas* | Soil |
| *Actinomyces* | Human | *Geodermatophilus* | Soil | *Pantoea* | Plant |
| *Actinophytocola* | Soil | *Gp6* | Non-bacterial | *Pelomonas* | Soil |
| *Actinotalea* | Soil | *Granulicatella* | Human | *Peptoniphilus* | Human |
| *Actinotignum* | Human | *Haemophilus* | Human | *Povalibacter* | Plant |
| *Aerococcus* | Human | *Haliscomenobacter* | Environment | *Prevotella* | Human |
| *Anaerococcus* | Human | *Herbaspirillum* | Plant | *Prosthecobacter* | Environment |
| *Anaerosphaera* | Human | *Hydrogenophaga* | Soil | *Pseudomonas* | Human/environment |
| *Aquabacterium* | Water | *Hydrotalea* | Water | *Pseudonocardia* | Water/environment |
| *Armatimonas* | Aquatic | *Kocuria* | Food | *Ralstonia* | Soil/environment |
| *Arthrobacter* | Soil | *Kosakonia* | Environment | *Ramlibacter* | Soil |
| *Azospirillum* | Soil | *Lactobacillus* | Food | *Rheinheimera* | Soil |
| *Bacillus* | Environment/human | *Lactococcus* | Food | *Rhodococcus* | Environment |
| *Bergeyella* | Human | *Lautropia* | Human | *Rubrivivax* | Environment |
| *Blastococcus* | Water/environment | *Leptotrichia* | Human | *Rudanella* | Environment |
| *Bordetella* | Human | *Leuconostoc* | Plant | *Ruminococcus* | Human |
| *Brevibacterium* | Environment/mammal | *Limnobacter* | Water | *Salmonella* | Food |
| *Campylobacter* | Food | *Lysinibacillus* | Soil/environment | *Selenomonas* | Human |
| *Cellvibrio* | Soil | *Lysobacter* | Soil | *Snodgrassella* | Insect |
| *Comamonas* | Soil/environment | *Marmoricola* | Soil | *Solibacillus* | Air |
| *Corynebacterium* | Animal | *Massilia* | Soil/environment | *Spirillospora* | Environment |
| *Craurococcus* | Environment | *Meiothermus* | water | *Staphylococcus* | Animal |
| *Cryptosporangium* | Plant | *Methylobacterium* | Water | *Stenotrophomonas* | Soil/environment |
| *Dechloromonas* | Environment | *Methyloversatilis* | Water | *Streptococcus* | Human |
| *Dietzia* | Plant | *Microbacterium* | Soil/environment | *Tetrasphaera* | Water |
| *Domibacillus* | Soil | *Micrococcus* | Human | *Turicella* | Human |
| *Enhydrobacter* | Water | *Micromonospora* | Environment | *Vampirovibrio* | Algae |
| *Enterobacter* | Human/environment | *Mobiluncus* | Human | *Variovorax* | Soil |
| *Escherichia* | Human | *Mycobacterium* | Mammal | *Veillonella* | Human |
| *Exiguobacterium* | Water | *Neisseria* | Human |  |  |

**Table 2:** Published 16S rRNA gene sequences retrieved from GenBank and included in the *Anaplasma* phylogenetic analysis in this study.

| Accession number | Genus/Species | Strain/Isolate | Accession number | Genus/Species | Strain/Isolate |
| --- | --- | --- | --- | --- | --- |
| AF414870.1 | *Anaplasma ovis* | OVI | MF576175.1 | *Anaplasma* | ST Mymensingh |
| CP015994.1 | *Anaplasma ovis* | Haibei | MH762077.1 | *Anaplasma capra* | AK-Rm-429 |
| CP000030.1 | *Anaplasma marginale* | St. Maries | U03775.1 | *Anaplasma bovis* | NA |
| CP001759.1 | *Anaplasma centrale* | Israel | KU586025.1 | *“Candidatus* Anaplasma boleense” | WHANSP-48 |
| OQ909445 | *Anaplasma* | KNP2 platys-like | MG869522.1 | *Anaplasma* | Zhengxiaocun |
| KU686784.1 | *Anaplasma centrale* | Uganda | MG869519.1 | *Anaplasma* | Wangmang |
| MK814419.1 | *Anaplasma platys* | Apla1 | LC269823.1 | *Anaplasma* | ZAM dog-181 |
| CP046391.1 | *Anaplasma platys* | S3 | AY570538.1 | *Anaplasma* | SA dog-1108 |
| U54806.1 | *Anaplasma* | Omatjenne | OK560162.1 | *Anaplasma bovis-*like | 9100t |
| CP006618.1 | *Anaplasma phagocytophilum* | Dog2 | NR118489.1 | *Anaplasma odocoilei* | UMUM76 |
| KF843825.1 | “*Candidatus* Anaplasma camelii” | Camel 7 | MK814449.1 | *Anaplasma* | Mymensingh |
| KF843824.1 | “*Candidatus* Anaplasma camelii” | Camel 4 | LC558313.1 | *Anaplasma* | Om5 |
| MK575506.1 | *Anaplasma* | Mongolia | NR074513.2* | *Ehrlichia ruminantium* | Welgevonden |

* Outgroup

**Table 3:** Published 16S rRNA gene sequences retrieved from GenBank and included in the *Coxiella* phylogenetic analysis in this study. *Coxiella*-like endosymbionts (CLE) are identified according to their tick host.

| Accession number | Species | Strain/ Isolate | Accession number | Species | Strain/ Isolate |
| --- | --- | --- | --- | --- | --- |
| CP011126.1 | *“Candidatus* Coxiella mudrowiae” | CRt | KP994833.1 | CLE *Rhipicephalus decoloratus* | Rhdeco1 |
| CP024961.1 | *“Candidatus* Coxiella mudrowiae” | CRS-CAT | KP994835.1 | CLE *Rhipicephalus evertsi* | Rhever1 |
| KU892220.1 | CLE *Rhipicephalus sanguineus* | NA | KP994839.1 | CLE *Rhipicephalus microplus* | Rhmicro1 |
| CP000890.1 | *Coxiella burnetii* | RSA 331 | KP994824.1 | CLE *Ixodes hexagonus* | Ihexa2 |
| MN263245.1 | *Coxiella burnetii* | B10 | KP994826.1 | CLE *Ixodes ricinus* | Iric2 |
| KC170759.1 | CLE *Haemaphysalis obesa* | TPSD8 | KP994795.1 | CLE *Ornithodoros rupestris* | Orupes1 |
| KP994843.1 | CLE *Rhipicephalus sanguineus* | Rhsa1 | KP994816.1 | CLE *Haemaphysalis punctata* | Haepun5 |
| KP994769.1 | CLE *Argas monachus* | Amo02 | KP994810.1 | CLE *Amblyomma variegatum* | Avar2 |
| KP994799.1 | CLE *Ornithodoros spheniscus* | Osphe1 | EF413063.1 | *Coxiella cheraxi* | TO-98 |
| KP994812.1 | CLE *Dermacentor marginatus* | Dmar2 | KP994804.1 | CLE *Amblyomma americanum* | Aame2 |
| KP994814.1 | CLE *Dermacentor silvarum* | Dsilv2 | U11021.1* | *Rickettsia rickettsii* | NA |
| MZ836861 | CLE *Rhipicephalus sanguineus* | no3-51 |  |  |  |

* Outgroup

**Table 4:** Published 16S rRNA gene sequences retrieved from GenBank and included in the *Ehrlichia/Wolbachia* phylogenetic analysis in this study.

| Accession number | Species | Strain/ Isolate | Accession number | Species | Strain/ Isolate |
| --- | --- | --- | --- | --- | --- |
| AB074459.1 | *“Candidatus* Ehrlichia shimanensis” | TS37 | EU833482.1 | *Wolbachia radopholus* | NA |
| KY425523.1 | *“Candidatus* Ehrlichia sp.” | Y272 | KX155505.1 | “*Candidatus* Wolbachia massiliensis” | PL13 |
| NR074513.2 | *Ehrlichia ruminantium* | Welgevonden | DQ402518.1 | “*Candidatus* Wolbachia inokumae” | NA |
| NR044747.1 | *Ehrlichia ewingii* | Stillwater | DQ402520.1 | “*Candidatus* Wolbachia brouquii” | NA |
| NR074500.2 | *Ehrlichia chaffeensis* | Arkansas | JX669531.1 | Uncultured bacterium | S3RP2010C1 |
| NR025962.1 | *Ehrlichia muris* | AS145 | KX155506.1 | *Wolbachia pipientis* | a.alb6-Marseille |
| DQ647318.1 | *Ehrlichia* sp. | HF | MN383047.1 | *Wolbachia* endosymbiont *Aedes aegypti* | WSF1-1 |
| NR148800.1 | *Ehrlichia minasensis* | UFMG-EV |  |  |  |
| NR118741.1 | *Ehrlichia canis* | Oklahoma | NR 043755.1* | *Rickettsia conorii raoultii* | Khabarovsk |
| MK507008.1 | *Ehrlichia canis* | CuD125 |  |  |  |
| JN121380.1 | Uncultured *Ehrlichia* | D28A |  |  |  |

* Outgroup

**Table 5:** Published 16S rRNA gene sequences retrieved from GenBank and included in the *Rickettsia* phylogenetic analysis in this study.

| Accession number | Species | Strain/ Isolate | Accession number | Species | Strain/ Isolate |
| --- | --- | --- | --- | --- | --- |
| DQ015802.1 | Uncultured bacterium | ELB16-030 | NR 074394.1 | *Rickettsia typhi* | Wilmington |
| NR 025967 | *Rickettsia honei* | RB | NR 044656.2 | *Rickettsia prowazekii* | Brein1 |
| KC331445.1 | Uncultured bacterium | lp227 | L36098 | *Rickettsia africae* | ESF-5 |
| MK616425.1 | Uncultured Rickettsiales | LG126_1 | U11021 | *Rickettsia rickettsii* | Sawtooth |
| MK616428.1 | Uncultured Rickettsiales | LG127_2 | NR 036848.1 | *Rickettsia sibirica* | 246 |
| MG563925.1 | “*Candidatus* Megaera benefice” | 1M-2 | NR 074469.2 | *Rickettsia conorii* | heilongjiangensis 054 |
| MG827267.1 | Uncultured *Rickettsia* | OTU389 | D38622 | *Orientia tsutsugamushi* | Gilliam |
| AF322442.1 | *Rickettsia limoniae* | Gent | D38626 | *Orientia tsutsugamushi* | Kuroki |
| NR 029155.1 | *Rickettsia canadensis* | 2678 | GU937608 | *Occidentia massiliensis* | Os18 |
| NR 074484.2 | *Rickettsia bellii* | RML369-C | NR149220 | *Occidentia massiliensis* | Os18 |
| NR 074459.2 | *Rickettsia japonica* | YH |  |  |  |
| NR 036773.1 | *Rickettsia australis* | NIAID Phillips 32 | NR 074500.2* | *Ehrlichia chaffeensis* | Arkansas |

* Outgroup

**Table 6:** Published 16S rRNA gene sequences retrieved from GenBank and included in the *Borrelia* phylogenetic analysis in this study.

| Accession number | Species | Strain/ Isolate | Accession number | Species | Strain/ Isolate |
| --- | --- | --- | --- | --- | --- |
| **Borrelia** | |  | NR 136451.1 | *Spirochaeta lutea* | JC230 |
| AB007450.1 | *Borrelia garinii* | ChY13p | NR 117123.1 | *Spirochaeta thermophila* | DSM 6578 |
| AJ224130.1 | *Borrelia burgdorferi* | CA446 | NR 044756.2 | *Spirochaeta halophila* | RS1 |
| EF570070.1 | *Borrelia* sp | SCW-41 | NR 043329.1 | *Spirochaeta dissipatitropha* | ASpC2 |
| MZ564265.1 | Uncultured *Borrelia* sp | K102 | AY599019.1 | *Spirochaeta aurantia* | M1 |
| AB113315.1 | *Borrelia duttonii* | VS4 | ***Trepnonema*** | |  |
| CP025785.1 | “*Candidatus* Borreliella tachyglossi” | Bc-F10-1268 | NR 181480.1 | *Treponema peruense* | RCC2812 |
| JF681792.1 | *Borrelia microti* | Abyek | NR 180741.1 | *Treponema phagedenis* | B43.1 |
| AF210137.1 | *Borrelia parkeri* | NA | NR 118740.1 | *Treponema pectinovorum* | 8:A:33768 |
| AY166715.1 | *Borrelia lonestari* | NA | ***Cristispira*** | |  |
| ***Spirochaeta*** | |  | U42638.1 | *Cristispira* | CP1 |
| NR 044505.1 | *Spirochaeta cellobiosiphila* | SIP1 |  |  |  |
| NR 134185.1 | *Spirochaeta psychrophila* | MO-SPC1 | NR043259.1* | *Leptospira borgpetersenii* | Veldrat Batavia 46 |
| NR 117137.1 | *Spirochaeta isovalerica* | DSM 2461 |  |  |  |

* Outgroup

**Table 7:** Summary of all samples from 2016, 2017, and 2019, providing information on raw sequence read counts, post-quality control read counts, and final read counts. Final read counts were calculated after chimeras, possible contaminants, and uncharacterized sequences were removed.

| Sample ID | Tissue Type | Raw read count | Post-quality control read count | Final read count |
| --- | --- | --- | --- | --- |
| Year: 2016 | | | | |
| Y1M3 | Midgut | 2002 | 1055 | 1055 |
| Y1M4 | Midgut | 3338 | 1804 | 1801 |
| Y1M6 | Midgut | 1709 | 1002 | 1002 |
| Y1M7 | Midgut | 1304 | 590 | 590 |
| Y1M8 | Midgut | 1291 | 643 | 643 |
| Y1M9 | Midgut | 3386 | 1618 | 1616 |
| Y1M10 | Midgut | 4758 | 2371 | 2369 |
| Y1S1 | Salivary gland | 6593 | 1842 | 1381 |
| Y1S2 | Salivary gland | 1820 | 913 | 910 |
| Y1S3 | Salivary gland | 864 | 428 | 428* |
| Y1S5 | Salivary gland | 1502 | 438 | 423* |
| Y1S7 | Salivary gland | 1972 | 905 | 905 |
| Y1S8 | Salivary gland | 1611 | 750 | 750 |
| Y1S9 | Salivary gland | 1990 | 1005 | 1000 |
| Y1S10 | Salivary gland | 4101 | 1663 | 1659 |
| Total read counts | | 38241 | 17027 | 16532 |
| Year: 2017 | | | | |
| Y2M1 | Midgut | 2699 | 1418 | 1295 |
| Y2M2 | Midgut | 997 | 605 | 599 |
| Y2M8 | Midgut | 1035 | 587 | 587 |
| Y2M9 | Midgut | 2741 | 1428 | 1422 |
| Y2S1 | Salivary gland | 1132 | 607 | 607 |
| Y2S2 | Salivary gland | 1075 | 627 | 625 |
| Y2S3 | Salivary gland | 1639 | 923 | 558 |
| Y2S4 | Salivary gland | 1354 | 746 | 743 |
| Y2S8 | Salivary gland | 1731 | 987 | 987 |
| Y2S10 | Salivary gland | 1239 | 628 | 601 |
| Total read counts | | 15642 | 8556 | 8024 |
| Year: 2019 | | | | |
| Y3M1 | Midgut | 9317 | 7509 | 4918 |
| Y3M3 | Midgut | 7882 | 6389 | 1374 |
| Y3M5 | Midgut | 6041 | 4823 | 1539 |
| Y3M6 | Midgut | 8544 | 7085 | 4400 |
| Y3M12 | Midgut | 200 | 153 | 70* |
| Y3M15 | Midgut | 6432 | 2911 | 273* |
| Y3S3 | Salivary gland | 2241 | 1646 | 268* |
| Y3S5 | Salivary gland | 2055 | 1543 | 715 |
| Y3S6 | Salivary gland | 2032 | 1504 | 1055 |
| Y3S10 | Salivary gland | 8083 | 6061 | 2225 |
| Y3S12 | Salivary gland | 9580 | 7763 | 1224 |
| Y3S15 | Salivary gland | 18288 | 7196 | 1155 |
| Total read counts | | 80695 | 54583 | 18605 |

*Low read count samples were removed from further analysis


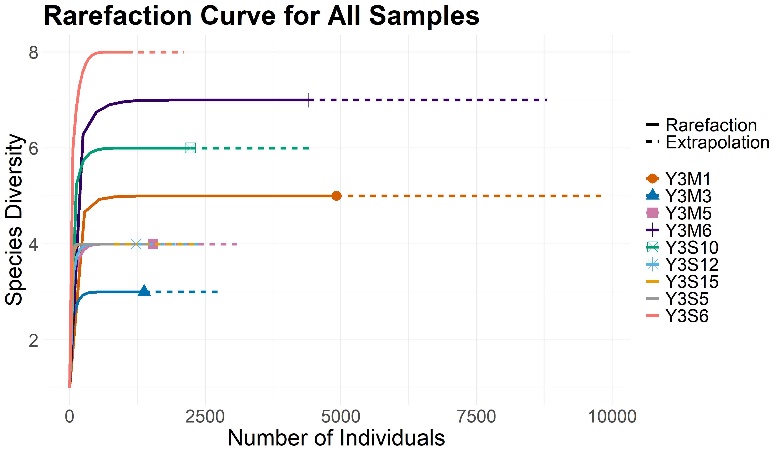

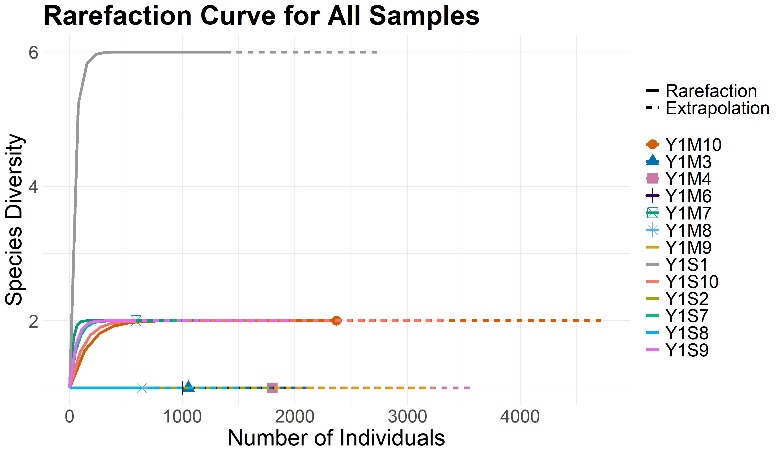

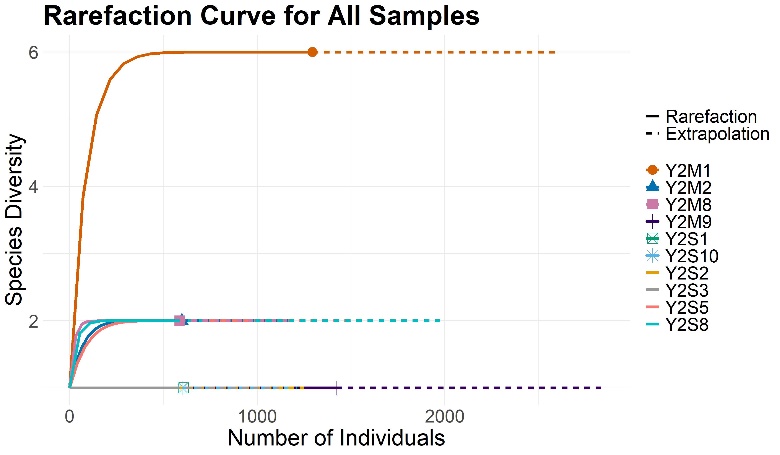


A

B

C

**Figure 1:** Rarefaction curves performed using the iNEXT package, for the bacterial microbiome data generated from midgut and salivay gland pools from *Rhipicephalus sanguineus* tick samples collected from dogs in (A) 2016, (B) 2017, and (C) 2019.


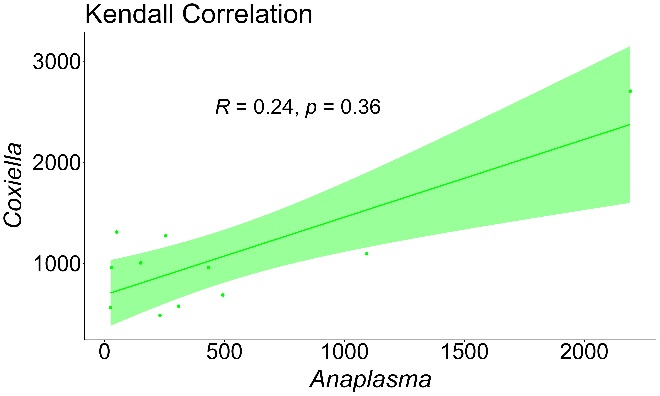

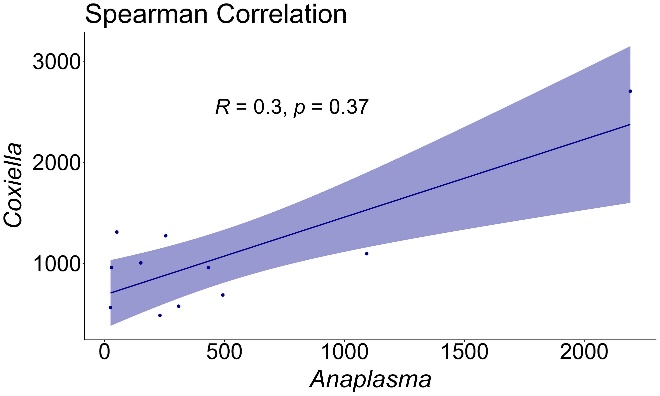


**Figure 2:** Kendall Correlation and Spearman Correlation tests indicating the association between the number of *Anaplasma* sequence reads and *Coxiella* sequence reads from the bacterial microbiome of *Rhipicephalus sanguineus* ticks collected from the study site in 2017 and 2019.


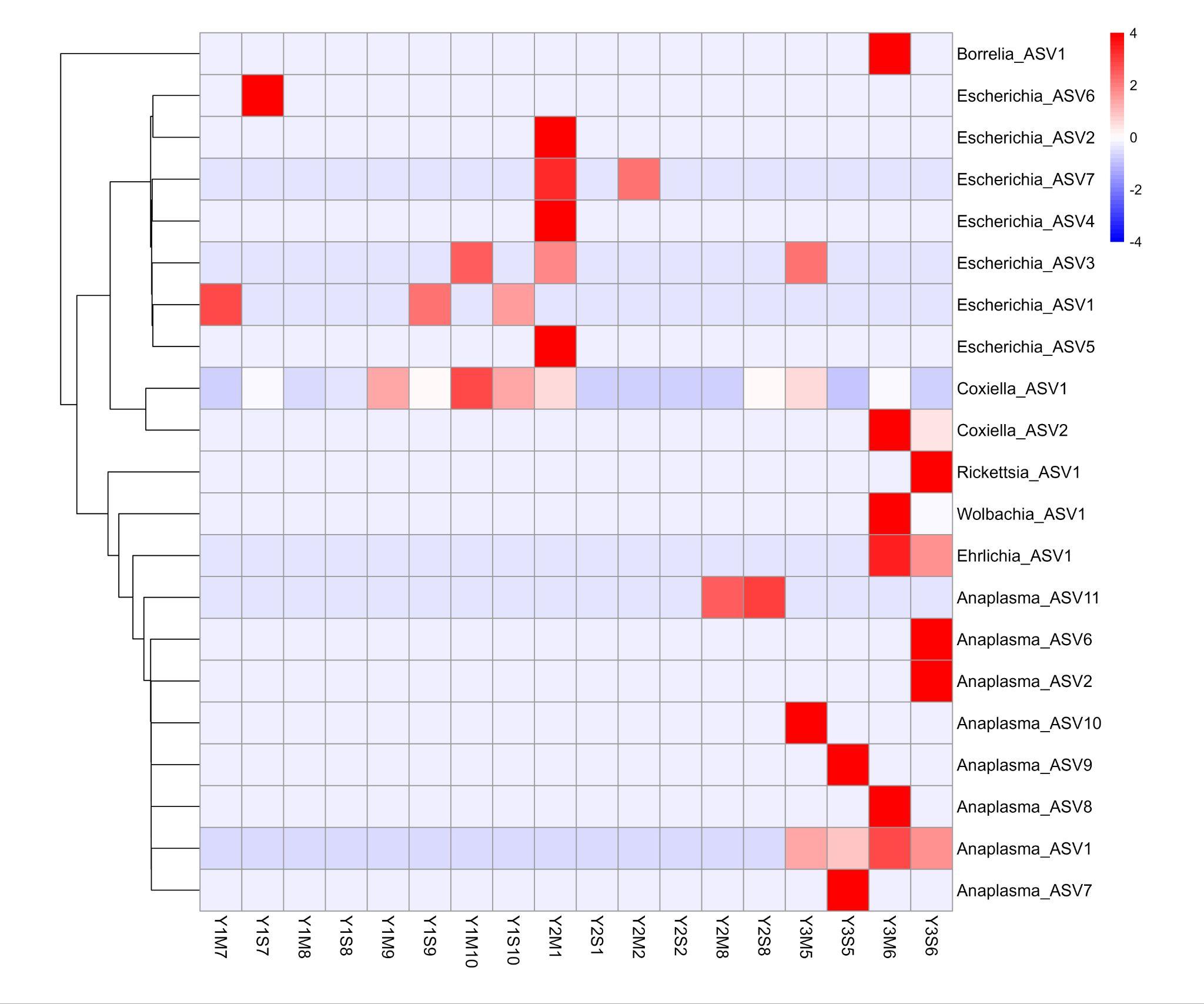


**Figure 3:** A heatmap visualizing differences in bacterial microbiome profiles of *Rhipicephalus sanguineus* s.l. midgut (M) and salivary gland (S) samples collected from dogs in Hluvukani, Bushbuckridge, Mpumalanga, South Africa, during 2016 (Y1), 2017 (Y2), and 2019 (Y3). The colour scale ranges from blue to red, where blue represents lower-than-average abundance and red indicates higher-than-average abundance, based on z-scores computed for each bacterial ASV. The dendrogram to the left of the heatmap reflects clustering based on pairwise alignment scores. Robust statistical testing could not be performed due to the small sample size.

**
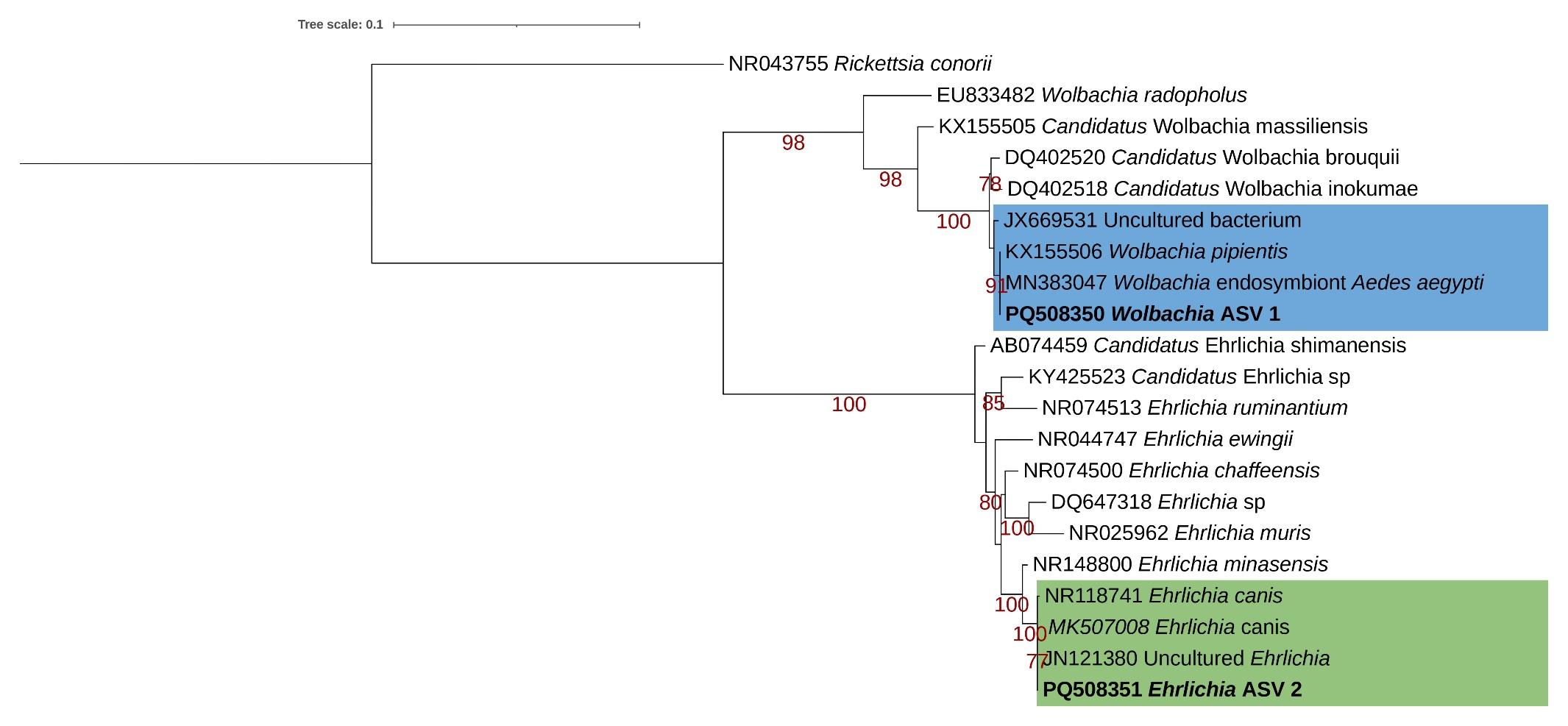
**

**Figure 4:** Maximum likelihood analysis indicating the relationships of *Ehrlichia* amplicon sequence variants detected in this study with known *Ehrlichia* and *Wolbachia* sequences from GenBank. A maximum likelihood inference was conducted using the TIM3+F+I+G4 model and 1000 bootstrap replicates (alignment length 1251 bp). Maximum likelihood bootstrap values (>75) are indicated below the branch. *Ehrlichia* amplicon sequence variants are indicated in bold. *Ehrlichia* sequences are indicated in a green box, while *Wolbachia* sequences are indicated in a blue box.

*
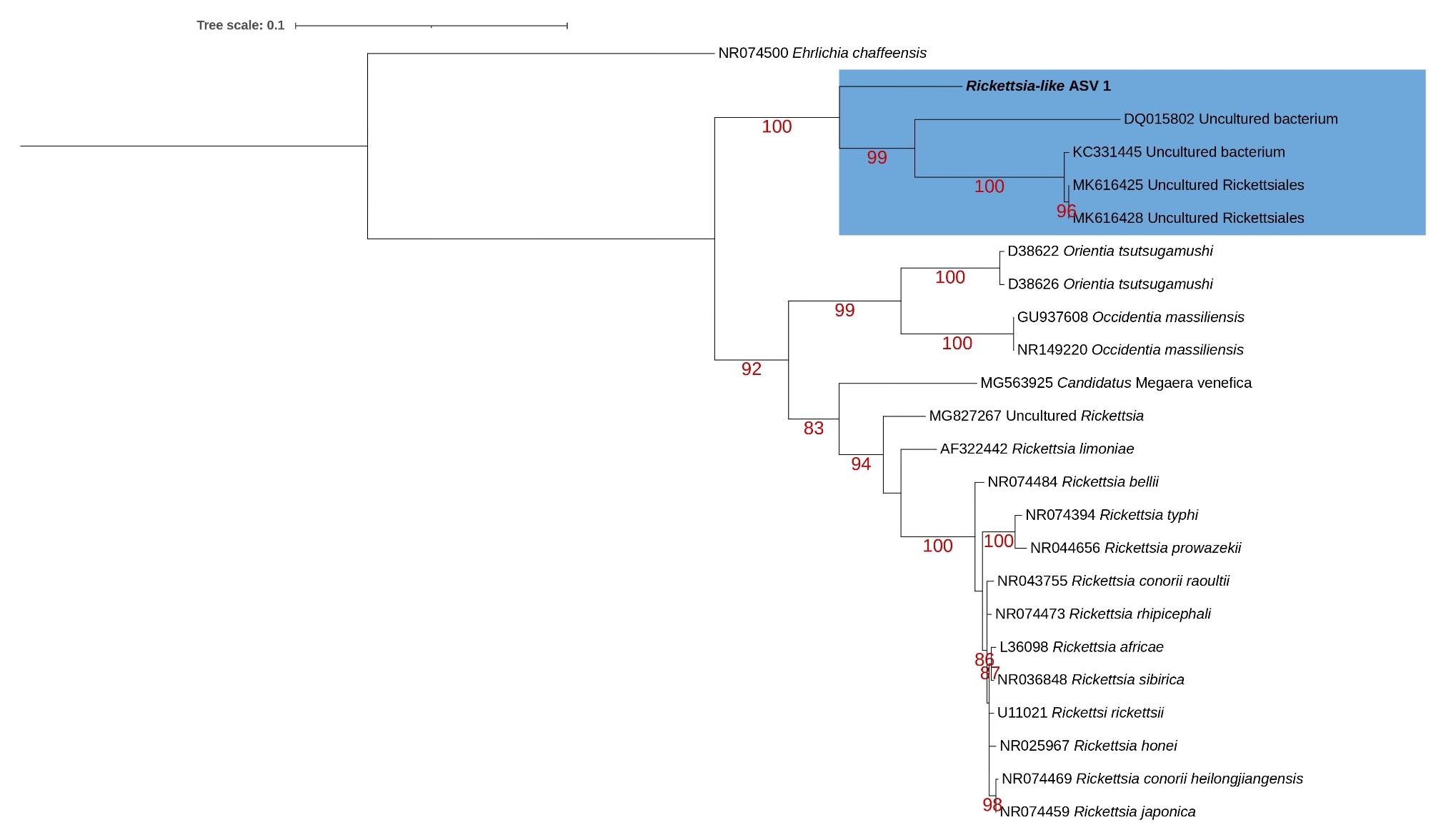
*

**Figure 5***:* Maximum likelihood analysis indicating the relationship of a *Rickettsia*-like amplicon sequence variant detected in this study with known *Rickettsia* sequences from GenBank. A maximum likelihood inference was conducted using the GTR+F+I+G4 model and 1000 bootstrap replicates (alignment length 1251 bp). Maximum likelihood bootstrap values (>75) are indicated below the branch. The *Rickettsia*-like amplicon sequence variant is indicated in bold. Uncultured *Rickettsiales* sequences are indicated in a blue box.


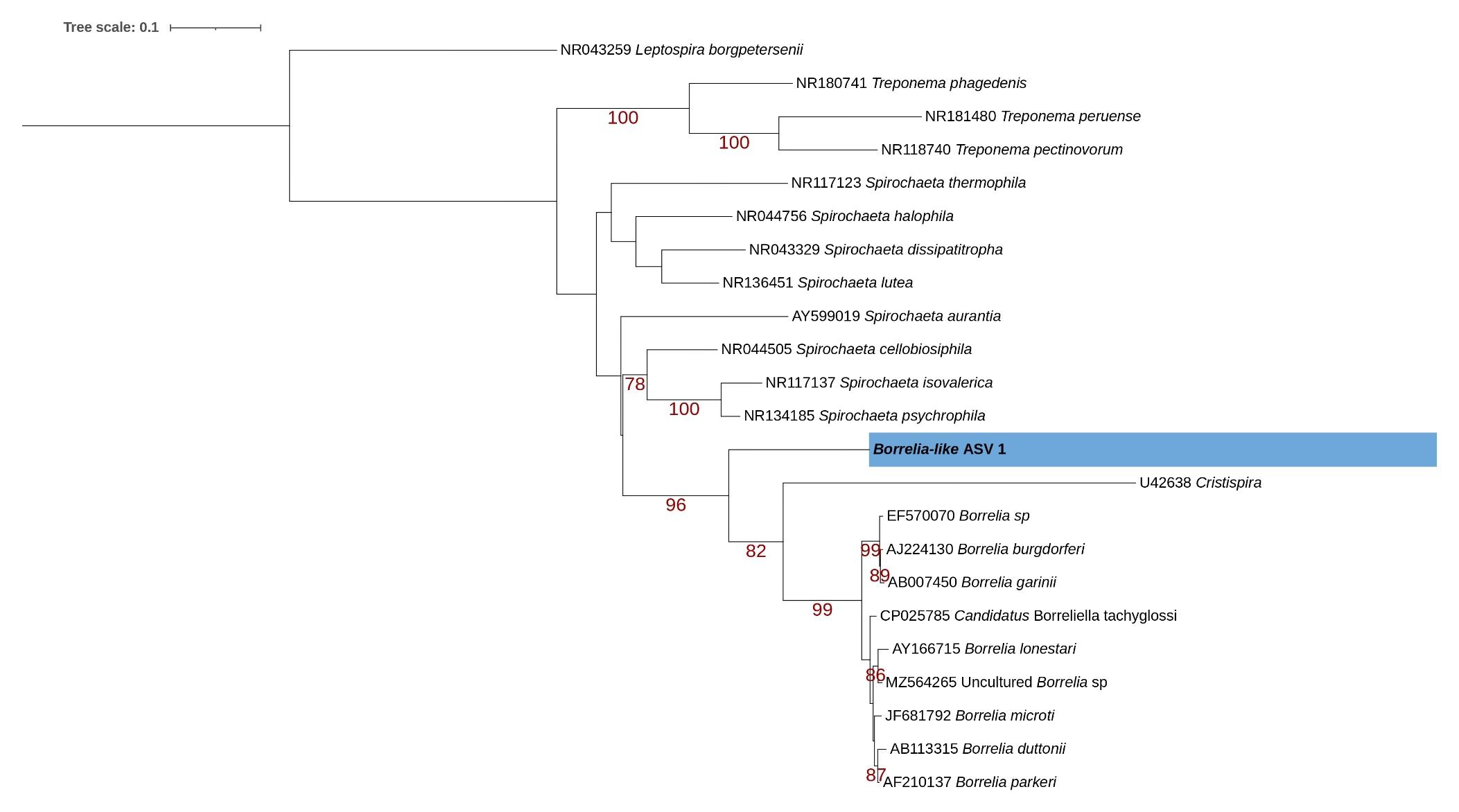


**Figure 6:** Maximum likelihood analysis indicating the relationship of a *Borrelia*-like amplicon sequence variant detected in this study with known sequences from taxa within the *Borreliaceae* family. A maximum likelihood inference was conducted using the TIM3+F+I+G4 model and 1000 bootstrap replicates (alignment length 1297 bp). Maximum likelihood bootstrap values are indicated below the branch. The *Borrelia*-like amplicon sequence variant is indicated in bold and highlighted in a blue box.
